# Heart Rate Variability Behavior during Exercise and Short-Term Recovery Following Energy Drink Consumption in Men and Women

**DOI:** 10.3390/nu12082372

**Published:** 2020-08-08

**Authors:** Nicolas W. Clark, Chad H. Herring, Erica R. Goldstein, Jeffrey R. Stout, Adam J. Wells, David H. Fukuda

**Affiliations:** 1 Physiology of Work and Exercise Response (POWER) Laboratory, Institute of Exercise Physiology and Rehabilitation Science, University of Central Florida, 12494 University Blvd., Orlando, FL 32816, USA; nicolas.clark@ucf.edu (N.W.C.); chad.herring@ucf.edu (C.H.H.); erica.goldstein@ucf.edu (E.R.G.); jeffrey.stout@ucf.edu (J.R.S.); 2 Division of Kinesiology, School of Kinesiology and Physical Therapy, University of Central Florida, 12494 University Blvd., Orlando, FL 32816, USA; adam.wells@ucf.edu; 3 Exercise Physiology Intervention and Collaboration (EPIC) Laboratory, Institute of Exercise Physiology and Rehabilitation Science, University of Central Florida, 12494 University Blvd., Orlando, FL 32816, USA

**Keywords:** energy drink, thermogenic drink formula, caffeine, autonomic nervous system, vagal withdrawal, heart rate variability threshold, sex-differences

## Abstract

This study examined the cardiac autonomic responses, as measured by heart rate variability (HRV), during cycling exercise and short-term rest after energy drink consumption. Seventeen participants (seven males and 10 females; age: 22.8 ± 3.5 years; BMI: 24.3 ± 3.3 kg/m^2^) completed this double-blind, placebo-controlled, counterbalanced crossover design study. Participants received an energy drink formula containing 140 mg of caffeine and a placebo in a randomized order before completing a 10-min steady-state warm up (WUP) and a graded exercise test to exhaustion (GXT) followed by a 15-min short-term rest (STR) period. Heartbeat intervals were recorded using a heart rate monitor. Data were divided into WUP, GXT, and STR phases, and HRV parameters were averaged within each phase. Additionally, root mean square of the standard deviation of R–R intervals (RMSSD) during GXT was analyzed to determine the HRV threshold. Separate two-way (sex (male vs. female) x drink (energy drink vs. placebo)) repeated measures ANOVA were utilized. Significant increases in high frequency (HF) and RMSSD were shown during WUP after energy drink consumption, while interactions between drink and sex were observed for HRV threshold parameters (initial RMSSD and rate of RMSSD decline). No significant differences were noted during STR. Energy drink consumption may influence cardiac autonomic responses during low-intensity exercise, and sex-based differences in response to graded exercise to exhaustion may exist.

## 1. Introduction

The consumption of energy drinks (ED), featuring caffeine as the primary active ingredient, has largely increased in the past two decades, and its market is expected to increase from 11 billion in 2018 to 83.4 billion in 2024 [1]. When consumed in moderate amounts (3–6 mg/kg), caffeine has the potential to aid performance during sport events [2,3]. Nevertheless, caffeinated drinks are also known to promote changes to the sympathovagal balance of the autonomic nervous system (ANS) [4], and overconsumption has been associated with adverse cardiac events such as cardiac arrhythmias and myocardial infarction [1,5]. Since vagal tone has an important cardioprotective role during exercise and recovery [6,7,8], and cardiovascular responses are exacerbated during exercise [9], further evaluation of ANS activity to stimulants contained in ED is necessary.

The time interval variation in a series of heartbeats is referred to as heart rate variability (HRV). Heart rate variability is a well-established biomarker that provides insight into ANS function, including cardiac parasympathetic nervous activity (cPNA) and cardiac sympathetic nervous activity (cSNA) [10,11]. While the influence of caffeine on HRV has recently been reviewed, it is still controversial in the literature due to the potential influence of caffeine tolerance [4]. Some evidence suggests that low doses of caffeine (100 and 200 mg) may decrease cPNA at rest in non-habitual caffeine users [12]; however, a more recent study showed no differences in HRV for the same amounts of caffeine while testing a group of habitual coffee drinkers who consumed one or more cups per day [13]. Alternatively, a previous study from our laboratory showed maintenance of vagal tone at rest with time-release caffeine supplementation (194 mg), and vagal withdrawal during placebo trials in a group of habitual caffeine consumers with a reported daily average intake greater than 200 mg [14].

In a study that examined differences in HRV following caffeine consumption (300 mg) vs. placebo among habitual caffeine consumer males during low-intensity endurance exercise below the ventilatory threshold (VT) [15], frequency domain outcomes including high frequency (HF), low frequency (LF), and total power (TP) resulted in a two-fold increase after caffeine ingestion, further suggesting that caffeine may stimulate the ANS response during exercise. To our knowledge, no experimental exercise studies have compared differences in ANS behavior during and post-exercise following a typical single caffeine intake (<200 mg) for habitual consumers [16], while including and comparing males’ and females’ responses. Although sex does not seem to affect hemodynamic and blood pressure responses to caffeine [17], significant changes in the ANS function as measured by HRV have been reported between sexes [18]. On average, females have greater HF and lower LF and TP for some metrics of frequency domain variables, and higher root mean square of the standard deviation of R–R intervals (RMSSD), HR, and therefore lower intervals between heartbeats among time frequency variables [18].

The heart rate variability threshold (HRVT) is another biomarker that has recently gained popularity due to its feasibility and low cost without the requirement of respiratory gas exchange or blood lactate analyses. It has also been suggested that this threshold mirrors metabolic changes during exercise and correlates with VT and LT [19,20,21]. As caffeine is effective at enhancing exercise performance [3], reducing the rating of perceived exertion [22], and influencing lactate levels during exercise [23,24], ED consumption could potentially influence the HRVT. The determination of the HRVT during incremental exercise requires identification of the point at which there is no further decline in HRV parameters, thus indicating vagal withdrawal [25]. Several methods of obtaining HRVT have been suggested for both time and frequency domains [20,21]. A commonly employed method involves visual inspection of the analyzed data by two or more researchers; however, this method is likely to present bias, and reliable evaluation is problematic. Therefore, standardized mathematical modeling have been used to capture HRV behavior during exercise [26].

The purpose of this study was to examine the influence of an ED containing 140 mg of caffeine on changes in ANS activity using HRV parameters during a low-intensity steady-state warm-up (WUP), a graded exercise to exhaustion test (GXT), and for 15 min of short-term rest post-exercise (STR) in a cohort of participants with regular caffeine intake. A secondary purpose was to examine HRV behavior, as measured by HRVT analysis, during the GXT alone. A tertiary purpose was to evaluate the potential for sex-based differences in both analyses. We hypothesized that the tested ED formula would increase ANS response and affect HRV variables during low-intensity exercise as well as incremental exercise to exhaustion and rest interval. We also expected that female participants would present higher cPNA responses when compared to males.

## 2. Materials and Methods

### 2.1. Experimental Design

Three visits to the laboratory scheduled in the morning were separated by at least 48 h and completed within two weeks. Participants were randomized to receive an energy drink (ED; 10 kcal, 296 mL drink containing a total of 140 mg of caffeine from a proprietary blend of caffeine, guarana, ginger, and green tea extract containing epigallocatechin gallate (EGCG)) or a placebo (PL; non-caloric/non-caffeinated, 296 mL artificially sweetened drink matching ED in taste and in color) in a double-blind, crossover fashion. Following the consumption of each drink, participants remained in a resting state, mostly in supine position, in the laboratory (controlled temperature of 21–24 °C) for approximately two hours before initiating the WUP and GXT on an electromagnetically-braked cycle ergometer (Corival, Lode B.V., Groningen, the Netherlands) while R–R intervals were continuously monitored using a heart rate monitor (Polar H10, Polar Electro Oy, Kempele, Finland). Prior to the GXT, participants completed a 10-min WUP at 50 watts for male participants and 30 watts for female participants. Immediately following the WUP, participants completed the GXT where work rate was increased by 35 watts for males and 25 watts for females every 3 min at a cadence between 70 and 80 rpm until volitional fatigue. After completing the GXT, participants remained seated on a chair for a 15-min period of short-term post-exercise recovery (STR). During STR, no external stimuli (e.g., conversations, smartphone, tablet, computer) were permitted and HRV continued to be monitored. This investigation was part of a larger study examining energy expenditure at rest and during exercise; however, the analyses presented focuses specifically on a comparison between two trials (one supplementation and one placebo) in order to highlight the effects of the 140 mg ED formula consumption on HRV data that have not been previously published [27].

### 2.2. Subjects

Data for 17 participants were retrieved and analyzed, including seven men (age = 24.9 ± 4.7 years, body mass = 73.2 ± 11.8 kg, body mass index = 25.7 ± 4.1 kg/m^2^) and 10 women (age = 21.3 ± 1.3 years, body mass = 64.6 ± 9.0 kg, body mass index = 23.3 ± 2.3 kg/m^2^). All participants were considered recreationally-active and met the American College of Sports Medicine standards of exercising ≥ 150 min exercise per week for the past 6 months [28] and were regular and moderate caffeine consumers (males = 140 ± 63 mg/day, females = 100 ± 43 mg/day), as estimated by a caffeine consumption questionnaire adapted from Landrum [29]. They were asked to refrain from ingesting caffeine or alcohol, to keep their diets consistent, and to avoid intense exercise for at least 24 h before testing days and to report to the laboratory following an 8-h fast.

The study protocol complied with the Declaration of Helsinki for human experimentation and was approved by the university’s ethical committee for human research (protocol number BIO-17-13679). This study was registered with ClinicalTrials.gov under the identifier NCT04455009.

### 2.3. HRV Parameters Analysis

The R–R series were recorded using a smartphone application (Elite HRV) and later downloaded and analyzed using a laptop with commercially available HRV analysis software (Kempele, Finland; Kubios HRV Analysis v 3.3, Kuopio, Finland). The software’s automatic artifact correction algorithm was used to detect artifacts from the R–R interval series and to separate and correct ectopic and misplaced beats from the normal sinus rhythm by interpolated R–R values. In all the subjects, and for each trial, the replaced R–R periods did not exceed 5% of total heartbeats. The R–R intervals were divided and averaged for WUP, GXT and STR phases. Each phase was analyzed for time domain values of mean R–R intervals (RR), root mean square of the standard deviation of R–R intervals (RMSSD), and maximal heart rate (HR_max_), minimum heart rate (HR_min_), and average heart rate (HR_mean_). Frequency domain analyses were estimated using Fast Fourier Transforms for high frequency (HF, HF%; 0.15–1.8 Hz), low frequency (LF, LF%; 0.04–0.15 Hz), and LF/HF ratio, and total power (TP). Additionally, breathing frequency (R*_f_*) data were collected using a facemask and metabolic gas analyzer (K-5 CPET, Cosmed, Rome, Italy).

### 2.4. HRVT Determination

**Figure 1:**
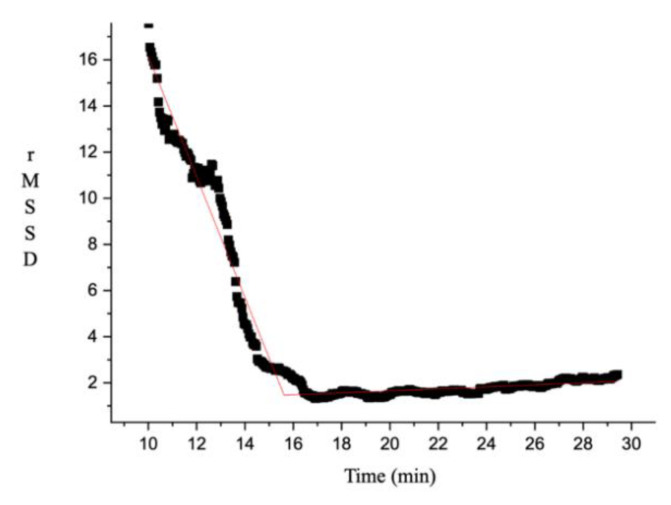
Example of a pairwise bilinear fitting function used to evaluate the heart rate variability threshold (HRVT). This analysis consists of two linear segments (solid red lines; R-squared = 0.98) used to perform a fit of the root mean square of the standard deviation of R–R intervals (RMSSD) moving average data (black marks) to calculate the intersection location for two linear segments from the fitting result.

Time domain analysis of RMSSD during the GXT was calculated using a time-varying method with 64-s moving windows and a 3-s shift [30]. A piecewise bilinear fitting function using a proprietary computer program for interactive scientific graphing (OriginPro, Version 2018b, OriginLab Corporation, Northampton, MA, USA) was used to evaluate RMSSD behavior and to determine HRVT during the GXT. Figure 1 exemplifies the pairwise bilinear fitting function. Measures for RMSSD at the beginning of the GXT (y-intercept of the first linear function), RMSSD decline rate (the slope of the first linear function), the time to HRVT and estimated time to vagal withdrawal (x-value at the intersection of the first and second linear functions), and RMSSD value at HRVT and its estimated value at vagal withdrawal (y-value at the intersection of the first and second linear functions) were recorded. R-squared was reported as a measure of goodness-of-fit for the piecewise bilinear fitting function (M ± SD).

### 2.5. Statistical Analysis

All analyses were conducted with an open-source statistical analysis software program (JASP; version 0.11). Alpha level was set a priori at *p* < 0.05. Median absolute deviation (MAD) was used to detect outlying values plus or minus two and a half times the MAD [31]. Outlying values were deleted before statistical analysis. The normality of the distribution was established using the Shapiro–Wilk statistic. In case of normality assumption violation, HRV data were transformed using a natural logarithm (Ln) prior to further statistical analysis. Two-way (sex (male vs. female) x drink (ED vs. PL)) repeated measures ANOVAs were used to compare ED vs. PL trials during WUP, GXT, and STR for RR, RMSSD, HR_max_, HR_min_, HR_mean_, HF, LF, LF/HF ratio, TP, and breathing frequency (R*_f_*). Two-way (sex (male vs. female) x drink (ED vs. PL)) repeated measures ANOVAs were also used to evaluate differences in RMSSD behavior during GXT between ED and PL for initial RMSSD, rate of RMSSD decline, HRVT, and RMSSD at HRVT. If a significant difference (*p* < 0.05) was observed, Holm post hoc analyses were conducted. Effect sizes were calculated as Cohen’s d values.

## 3. Results

### 3.1. Effects of Energy Drink on HRV Parameters during Warm-Up, Graded Exercise Test, and Short-Term Rest

**Figure 2:**
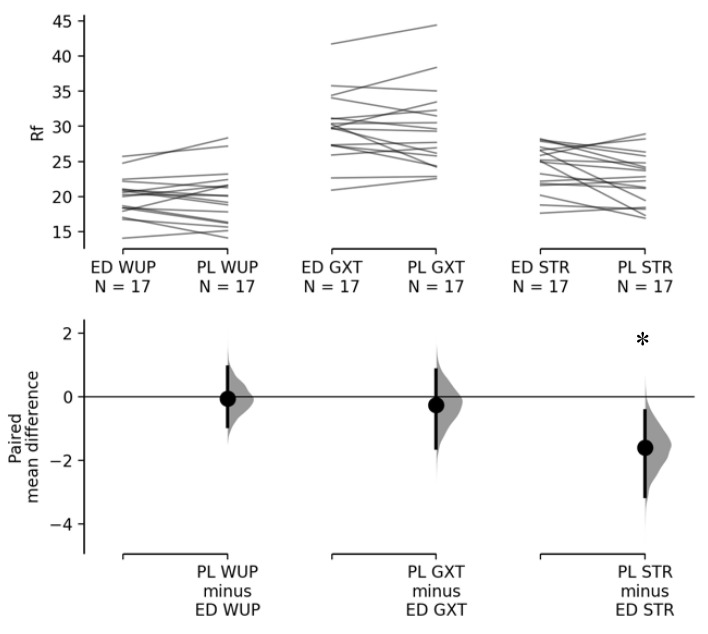
Paired mean difference for breathing rate (R*_f_*; breaths per min) during the warm-up (WUP), graded exercise test (GXT), and short-term rest post exercise (STR) are shown above in the Cumming estimation plot. The raw data are plotted on the upper axes; each paired set of energy drink (ED) and placebo (PL) is connected by a line. On the lower axes, each paired mean difference is plotted as a bootstrap sampling distribution. Mean differences are depicted as dots; 95% confidence intervals are indicated by the ends of the vertical error bars [32]; * Significant differences between drinks (*p* < 0.05).

Marginal means for drink ± SE, main effects for sex and drink, and interactions between sex x drink for frequency domain and time domain variables can be found in Table 1 and Table 2, respectively. Significant sex x drink interactions were shown for LnHF and HF% during the GXT; however post-hoc comparisons did not detect significant differences for LnHF (*p* > 0.05; ED_female_ = 2.50 ± 0.31; ED_male_ = 2.71 ± 0.34; PL_female_ = 2.08 ± 0.28; PL_male_ = 3.16 ± 0.39) and HF% (ED_female_ = 43 ± 4%; ED_male_ = 31 ± 5%; PL_female_ = 35 ± 3%; PL_male_ = 34 ± 5%). Significant sex x drink interactions were also shown during the GXT for LF/HF with no significant post-hoc differences (ED_female_ = 1.44 ± 0.28; ED_male_ = 1.99 ± 0.42; PL_female_ = 1.55 ± 0.21; PL_male_ = 1.70 ± 0.27). A significant main effect for sex was shown for LF% during the WUP (female = 46 ± 4%; male = 60 ± 4%) and for LnLF during the GXT (female = 2.53 ± 0.23; male = 3.49 ± 0.23). No significant interactions were shown for R*_f_* during the WUP, GXT, or STR; however, significant main effects were shown for drinks during the STR (Figure 2). Moreover, significant main effects for sex were shown for R*_f_* during the STR (*p* = 0.015; females = 22 ± 1 breaths per min; males = 25 ± 1 breaths per min).

### 3.2. Effect of Energy Drink on HRVT

There were no significant sex x drink interactions for HRVT (time value at the intersection of the first and second linear function; *p* = 0.246). No main effects were shown for drink (*p* = 0.149) or sex (*p* = 0.667; ED_Female_ = 5.65 ± 2.73 min; ED_Male_ = 6.96 ± 2.56 min; PL_Female_ = 5.43 ± 2.13 min; PL_Male_ = 5.11 ± 2.80 min). Similarly, the RMSSD value at the HRVT also did not present significant sex x drink interactions (*p* = 0.269). No significant main effects were noted for drink (*p* = 0.663) and sex (*p* = 0.628; ED_Female_ = 2.34 ± 0.52 ms; ED_Male_ = 2.24 ± 0.50 ms; PL_Female_ = 2.21 ± 0.42 ms; PL_Male_ = 2.52 ± 0.7676 ms). Nonetheless, significant sex x drink interactions were shown for initial RMSSD (*p* = 0.038) and rate of RMSSD decline (*p* = 0.039). However, no significant post-hoc comparisons were noted between drinks and sexes for either initial RMSSD (ED_Female_ = 29.92 ± 20.08 ms; ED_Male_ = 21.53 ± 9.42 ms; PL_Female_ = 25.17 ± 9.65 ms; PL_Male_ = 33.11 ± 17.88 ms) or rate of RMSSD decline (ED_Female_ = −1.82 ± 1.33 ms/min; ED_Male_ = −1.12 ± 0.49 ms/min; PL_Female_ = −1.54 ± 0.72 ms/min; PL_Male_ = −2.03 ± 0.96 ms/min). The average R-squared value for the piecewise bilinear fitting function analyses was 0.90 ± 0.08.

## 4. Discussion

This study examined the influence of ED ingestion on the ANS as measured by HRV time and frequency domain parameters during WUP, GXT, and STR while also comparing responses between males and females that habitually consumed caffeinated products. Another aim of this study was to evaluate the influence of ED on HRVT during GXT in males and females. Our main findings indicated that an ED formula containing 140 mg of caffeine, guarana, ginger, and green tea extract containing EGCG was able to affect HRV variables during WUP, GXT, and STR, while sex-based differences may occur in HRVT during GXT.

In this study, the WUP consisted of 10-min steady-state cycling at a fixed light exercise intensity of approximately 20% of peak power achieved during the GXT. At this intensity, we noted significant changes in HRV responses after ED ingestion. For frequency domain variables, increases in LF, HF, and TP were observed for the ED drink compared to PL. These changes are thought to mirror enhancement in the ANS activity during this light activity [33]. More notably, increases in the HF band are suggestive of augmented cPNA. Similar changes to frequency domain variables were reported by Nishijima et al. [15] when evaluating a group of habitual caffeine consumers cycling at slightly higher work rates for 30 min (60–80 W). Possible increased cPNA was also displayed by a higher RMSSD for the ED, which is the time domain marker widely used for the evaluation of short term vagally mediated changes in HRV [34]. In habituated individuals, caffeine has been suggested to sustain and promote cPNA at rest conditions [14]. Furthermore, an increased heart rate maximum (HR_max_) for the ED was observed. Interestingly, this change in HR_max_ during low-intensity aerobic exercise has not been previously reported following ED consumption. The last observable change during the WUP related to sex differences for LF% with females showing lower relative values. This finding is consistent with reported sex differences in HRV spectral power responses during exercise [35], which may be explained by altered ANS function and hormonal differences [18].

Another important aspect of this study was to understand the effect of ED ingestion on HRV behavior during a maximal GXT. As the main active component of ED, caffeine has been suggested to influence the cardiovascular system via the amplification of cSNA, resulting in increases in heart rate and blood pressure [36]. Additional ingredients in ED formulas could influence HRV responses even further. These effects on the cardiovascular system have been a reason for public concern due to the increased possibility of adverse events in certain populations [37]. Moreover, vagal tone has a cardioprotective role during rest and exercise [8]; therefore, understanding the effect of ED on vagal withdrawal estimation was an important part of this study. We used a pairwise bilinear fitting function to analyze the withdrawal (HRVT) and to specifically define the time point associated with the termination of RMSSD time varying analysis decline during exercise. Our results show that HRVT was not affected by ED ingestion when compared to PL. It is possible that the tested caffeine concentration of 1.96 ± 0.36 mg/kg for males and 2.21 ± 0.34 mg/kg for females might not result in noticeable changes in the estimated time to vagal withdrawal during exercise. Nevertheless, an interesting finding from HRVT analysis was that female participants displayed a lower RMSSD output at the beginning of GXT testing during PL, but a higher value during ED when compared to men. However, post-hoc comparisons failed to detect specific changes in this interaction. This finding is partly supported by a significant interaction between drink and sex for LF/HF and HF% averages during the GXT, further suggesting that males and females might respond differently to ED during exercise. Sex differences were also shown for LF during the GXT. Simultaneously, time domain averaged values during GXT showed decreases in mean R–R intervals and increased HR_max_ and HR_min_ averages for the ED.

Heart rate variability parameters post-exercise have been increasingly studied after consumption of caffeine and energy drinks [4,36,38]. There seems to be an agreement that the suppression of cPNA reactivation post-exercise can be unfavorable for increased susceptibility of a cardiac event [8]. While our results point to a decreased mean RR and a trend for decreased HF during STR after ED consumption, we believe that this may not be indicative of a delayed or reduced cPNA reactivation. Rather, the respiratory sinus arrhythmia, one of the most conspicuous variables known to affect HRV—but not necessarily the ANS—might have influenced this outcome [34]. The HF band is also termed the respiratory band due to the influence that breathing can play in HF modulation [39]. Spirometry results recorded during testing showed a significant increase for breathing frequency for the ED during STR post-exercise, but not for WUP and GXT (Figure 2). This significant increase in the breathing rate is thought to have influenced some of the outputs and, therefore, should not be considered as a potential alteration in the ANS function but a product of respiratory sinus arrhythmia. Moreover, it is important to note that RMSSD was not significantly altered during STR. Previous studies have shown that RMSSD is comparatively more robust and less susceptible to breathing pattern changes than HF [40], which could explain the comparable findings in the current study.

Several limitations to this study should be considered. While the results from the current study may potentially be generalized to the absolute dosage of caffeine, the specific contributions of the individual ingredients contained within the proprietary ED formula were not evaluated. Moreover, it is important to note that absolute doses of caffeine were tested in this investigation and relative intake per kg of body mass was not controlled. Nonetheless, there were no statistical differences between males and females for 140 mg (*p* = 0.167). Lastly, we did not control for the menstrual cycle phase when scheduling visits, and this could have potentially altered HRV outputs for female participants [41].

## 5. Conclusions

From this investigation, we conclude that ED containing 140 mg of caffeine (caffeine, guarana, ginger, and green tea extract containing EGCG did not result in decreased cPNA as measured HRV-specific parameters within this cohort of habituated caffeine consumers. In fact, the opposite was true during low-resistance steady-state WUP exercise, although significantly increased HRs were found during WUP, GXT, and STR. Sex-based differences were displayed during WUP and GXT, while an interaction was shown between males and females for HRVT parameters during the GXT. Furthermore, consumption of the tested ED formula did not influence HRVT during GXT. Lastly, no significant changes in ANS function were shown after exercise. Future studies should seek to compare individuals of different caffeine consumption status on HRV responses during and after exercise.

## Figures and Tables

**Table 1:** Comparison of frequency domain parameters during warm up, graded exercise test, and short-term recovery 15 min post exercise.

		*df*	Energy Drink (ED) M ± SE	Placebo (PL) M ± SE	Within Drink ED vs. PL *p-Value* *(Cohens-d)*	Between Sex Male vs. Female *p-Value* *(Cohens-d)*	Interaction Drink x Sex *p-Value*
*Warm up*	LnHF	14	5.65 ± 0.17	4.95 ± 0.17	<0.001 * (1.731)	0.799	0.781
	HF%	14	41 ± 3	39 ± 3	0.592	0.111	0.655
	LnLF	15	5.91 ± 0.18	5.42 ± 0.18	<0.001 * (1.098)	0.630	0.688
	LF%	15	52 ± 33	54 ± 3	0.584	0.049 * (0.519)	0.832
	LF/HF	13	0.24 ± 0.19	0.37 ± 0.19	0.342	0.116	0.950
	LnTP	14	6.60 ± 0.17	6.08 ± 0.17	<0.001 * (1.500)	0.238	0.229
*Graded Exercise Test*	LnHF	15	2.55 ± 0.23	2.56 ± 0.23	0.937	0.149	0.038 *
	HF%	15	37 ± 3	35 ± 3	0.261	0.271	0.022 *
	LnLF	15	2.97 ± 0.19	3.05 ± 0.19	0.691	0.010 * (0.716)	0.258
	LF%	13	52 ± 3	52 ± 3	0.904	0.154	0.364
	LF/HF	15	0.42 ± 0.14	0.48 ± 0.14	0.532	0.252	0.049 *
	LnTP	14	3.55 ± 0.18	3.55 ± 0.18	0.998	0.069	0.208
*Short Term Rest*	LnHF	15	3.06 ± 0.34	3 ± 0.34	0.058	0.834	0.885
	HF%	12	14 ± 3	19 ± 3	0.059	0.086	0.180
	LnLF	15	4.59 ± 0.26	5.07 ± 0.26	0.078	0.442	0.819
	LF%	15	70 ± 3	68 ± 3	0.534	0.133	0.659
	LF/HF	14	1.51 ± 0.22	1.34 ± 0.22	0.151	0.218	0.741
	LnTP	14	4.94 ± 0.26	5.42 ± 0.26	0.096	0.725	0.973

* Significant differences (*p* < 0.05). HF—High Frequency; LF/HF—Low Frequency High Frequency Ratio; TP—Total Power.

**Table 2:** Comparison of time domain parameters during warm up, graded exercise test, and short-term recovery 15 min post exercise.

		*df*	Energy Drink (ED) M ± SE	Placebo (PL) M ± SE	Within Drink ED vs. PL *p-Value* *(Cohens-d)*	Between Sex Male vs. Female *p-Value* *(Cohens-d)*	Interaction Drink x Sex *p-Value*
*Warm up*	Mean RR (ms)	15	598 ± 17	610 ± 17	0.207	0.254	0.894
	HR_mean_ (bpm)	15	99 ± 3	99 ± 3	0.910	0.526	0.189
	HR_max_ (bpm)	14	115 ± 3	109 ± 3	0.008 * (0.770)	0.630	0.688
	HR_min_ (bpm)	13	75 ± 3	71 ± 3	0.217	0.798	0.859
	LnRMSSD	13	2.9 ± 0.1	2.7 ± 0.1	0.006 * (0.792)	0.671	0.999
*Graded Exercise Test*	Mean RR (ms)	15	397 ± 6	413 ± 6	0.005 * (-0.805)	0.065	0.515
	HR_mean_ (bpm)	15	155 ± 3	149 ± 3	0.016 * (0.685)	0.111	0.635
	HR_max_ (bpm)	14	190 ± 2	184 ± 2	0.014 * (0.702)	0.235	0.708
	HR_min_ (bpm)	14	106 ± 3	100 ± 3	0.028 * (0.613)	0.645	0.898
	LnRMSSD	14	1.44 ± 0.09	1.39 ± 0.09	0.451	0.773	0.079
*Short Term Rest*	Mean RR (ms)	13	509 ± 10	533 ± 10	0.023 * (-0.666)	0.400	0.915
	HR_mean_ (bpm)	15	118 ± 3	112 ± 3	0.021 * (0.628)	0.566	0.731
	HR_max_ (bpm)	15	187 ± 3	180 ± 3	0.041 * (0.541)	0.131	0.377
	HR_min_ (bpm)	15	94 ± 3	87 ± 3	0.009 * (0.728)	0.854	0.523
	LnRMSSD	11	1.63 ± 0.13	1.91 ± 0.13	0.104	0.546	0.673

* Significant differences (*p* < 0.05). Mean RR—mean differences in R–R intervals; HR_mean_—Mean Heart Rate; HR max—Maximum Heart Rate Achieved; HR_min_—Minimum Heart Rate Achieved; RMSSD—Root Mean Square of the Successive R–R intervals.
